# The relationship between lateral meniscus shape and joint contact parameters in the knee: a study using data from the Osteoarthritis Initiative

**DOI:** 10.1186/ar4455

**Published:** 2014-01-28

**Authors:** Kai Yu Zhang, Angela E Kedgley, Claire R Donoghue, Daniel Rueckert, Anthony MJ Bull

**Affiliations:** 1 Department of Bioengineering, Imperial College London, South Kensington Campus, London SW7 2AZ, UK; 2 Department of Computing, Imperial College London, South Kensington Campus, London SW7 2AZ, UK

## Abstract

**Introduction:**

The meniscus has an important role in force transmission across the knee, but a detailed three-dimensional (3D) morphometric shape analysis of the lateral meniscus to elucidate subject-specific function has not been conducted. The aim of this study was to perform 3D morphometric analyses of the lateral meniscus in order to correlate shape variables with anthropometric parameters, thereby gaining a better understanding of the relationship between lateral meniscus shape and its load-bearing function.

**Methods:**

The lateral meniscus (LM) was manually segmented from magnetic resonance images randomly selected from the Osteoarthritis Initiative (OAI) non-exposed control subcohort. A 3D statistical shape model (SSM) was constructed to extract the principal morphological variations (PMV) of the lateral meniscus for 50 subjects (25 male and 25 female). Correlations between the principal morphological variations and anthropometric parameters were tested. Anthropometric parameters that were selected included height, weight, body mass index (BMI), femoral condyle width and axial rotation.

**Results:**

The first principal morphological variation (PMV) was found to correlate with height (r = 0.569), weight (r = 0.647), BMI (r = 0.376), and femoral condyle width (r = 0.622). The third PMV was found to correlate with height (r = 0.406), weight (r = 0.312), and femoral condyle width (r = 0.331). The percentage of the tibial plateau covered by the lateral meniscus decreases as anthropometric parameters relating to size of the subject increase. Furthermore, when the size of the subject increases, the posterior and anterior horns become proportionally longer and wider.

**Conclusion:**

The correlations discovered suggest that variations in meniscal shape can be at least partially explained by the levels of loads transmitted across the knee on a regular basis. Additionally, as the size of the subject increases and body weight rises, the coverage percentage of the meniscus is reduced, suggesting that there would be an increase in the load-bearing by the cartilage. However, this reduced coverage percentage is compensated by the proportionally wider and longer meniscal horn.

## Introduction

The menisci are crescent-shaped intra-articular fibrocartilages located between femur and tibia. Meniscectomy persists as a treatment for meniscal tears, despite early work showing detrimental radiological changes post meniscectomy [1]. This has since been confirmed by many long-term follow-up studies [2-5]. Cadaveric work has shown significant increases in contact stress due to partial or total meniscectomies [6-8], suggesting that the meniscus has an important role in force transmission across the knee. Furthermore, the medial and lateral menisci appear to perform differently in both load bearing and load distribution. Although the medial compartment sustains more weight-bearing stress [7-9], the lateral meniscus (LM) covers a greater percentage of the area of its compartment than the medial meniscus [7]. There is also potentially more movement on the lateral tibia [10]. Therefore, the LM may potentially contribute more to load bearing in the lateral compartment than the medial meniscus does in the medial compartment [7,11]. Evidence of worse radiographic results and higher incidence of late osteoarthritis (OA) associated with lateral meniscectomy compared to medial meniscectomy may support this hypothesis [4,12-15], although the convexity of the tibial plateau might also be a factor.

The load-bearing ability of biological tissues is a function of geometry, movement, and deformation properties; the meniscus is a crucial load-bearing structure that minimises contact stress by the creation of hoop stresses, thus optimising contact area [7,8]. Therefore, the shape of the meniscus is especially important in loading. Recent three-dimensional stress analysis studies have shown the significance of insertional ligament geometry and meniscus material properties on their load-bearing capability [16]. However, a three-dimensional morphometric shape analysis of the LM in order to elucidate subject-specific function has not been conducted. The aim of this study was to perform three-dimensional morphometric analyses of the LM to discover correlations with anthropometric parameters and gain a better understanding of the relationship between LM shape and its potential load-bearing capability. The chosen anthropometric parameters included height, weight, body mass index (BMI), femoral condyle width and axial rotation of the knee.

Height, weight and BMI reflect the level of loading applied to the knee, and have previously been found to correlate with meniscus size and position [17-19] and OA; being overweight increases the risk of OA [20,21] and greater height has been reported as a risk factor for knee injuries [22]. Condylar width is a measure of knee size, and knee rotational position is a geometric variable that is not known to relate to contact stress; however, as the menisci are known to move with knee rotation, it was hypothesised that there is a correlation between rotational position and meniscus shape. BMI is correlated with knee overuse injury [23], acute knee injury and associated pathologies [24], and degenerative joint disease [23,25,26]; therefore, a correlation with BMI was also expected.

## Methods

### Study sample

Fifty subjects from the Osteoarthritis Initiative (OAI) were randomly selected from the “non-exposed” reference cohort (age 56 ± 8 years; height 169.1 ± 9.7 cm; weight 70.93 ± 11.98 kg; BMI 24.7 ± 3.0 kg/m^2^; 25 male and 25 female). The non-exposed cohort contains subjects for which there are no symptoms of OA present, no radiographic signs of knee OA, and no risk factors for developing knee OA. These inclusion criteria enable observation of normal meniscus function in subjects who are unlikely to develop OA. The data are available at [27].

This study used magnetic resonance (MR) images from the OAI database from groups 0.C.2 and 0.E.1 at baseline. The fat-suppressed, sagittal three-dimensional double-echo in steady state (DESS) sequence with water excitation (WE) (referred to here as Sag 3D DESS) was selected because it has both high in-plane resolution (0.365 × 0.365 mm) and a small slice-thickness (0.7 mm). Further information about the imaging protocol can be found in Peterfy *et al.*[28].

The axial rotation of the tibia with respect to the femur was quantified by determining the surgical epicondylar axis on the femur and the posterior condylar axis on the tibia, measured at 8 mm distal to the joint surface [29]. The anthropometric parameters of weight, height, BMI, and femoral condyle width were downloaded from the OAI dataset labelled *PhysExam00* and *kXR_QJSW_Duryea00* from release version 0.2.2; the variables are named P01WEIGHT, P01HEIGHT, P01BMI and V00CFWDTH. Ethical approval and informed consent were not required as the data used in this study was from a publically available dataset.

### Morphometric analysis of the lateral meniscus

**Figure 1:**
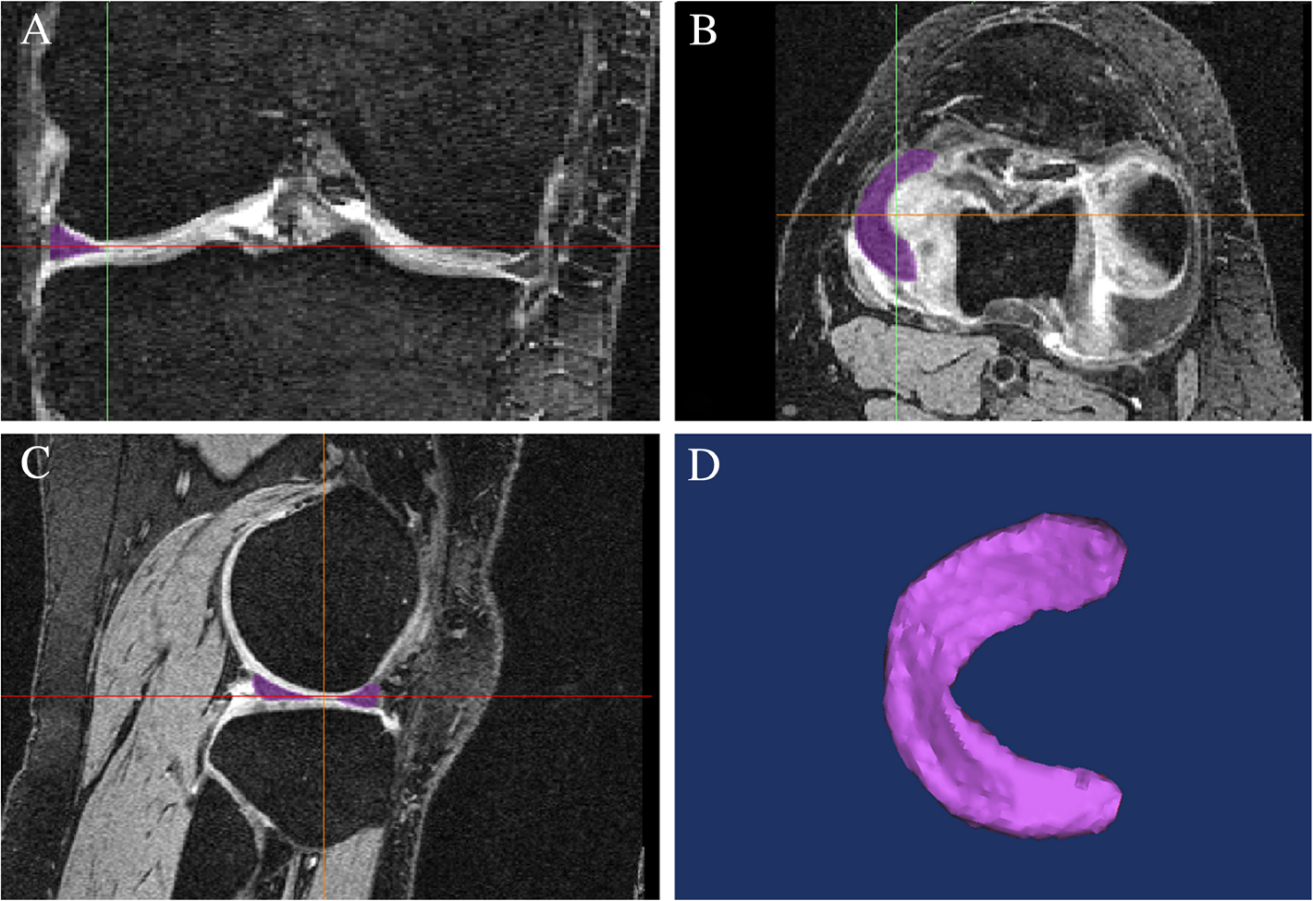
**Segmentation of the magnetic resonance MR images. (A)** Coronal plane; **(B)** sagittal plane; **(C)** transverse plane; **(D)** three-dimensional surface model.

The MR images were manually segmented from sagittal, coronal and transverse views using Mimics (Materialise NV, Leuven, Belgium) as can be seen in Figure 1. The segmentations were then smoothed using the *reduce-noise* option in Geomagic Studio (Geomagic, Inc., Morrisville, NC, USA) to achieve three-dimensional surface models. To minimise the interventions to the surface detail, a single iteration of noise reduction was used. The smoothness results indicated that throughout the 50 sets of surface models the average distance that all points were moved was between 0.008 and 0.016 mm.

Statistical shape modelling (SSM) as described by Zhang *et al.*[30] was used to perform the three-dimensional morphometric analysis and reveal the significant shape parameters. This is a model-based image analysis technique that aims to establish any linear patterns of variation in the shape and any spatial relationships between the structures in a given class of images.

The shape of a subject’s LM can be defined by a vector of point coordinates as follows:

$$\left(x=\left[x_{1},x_{2},\ldots x_{n},y_{1},y_{2},\ldots y_{n},z_{1},z_{2},\ldots z_{n}\right]\right)$$

where there are n surface points in the surface model. To compute the SSM, an LM surface model in the dataset was randomly chosen as the reference segmentation. All other three-dimensional surface models of the LMs were aligned to the coordinate system defined by the reference segmentation using the iterative closest-point algorithm [31]. Point-to-point correspondences were then established for each subject to the reference subject using the multi-resolution free-form deformation algorithm proposed by Rueckert *et al.*[32]. A mean model was computed for each set of corresponding points from each LM surface model (**x**), and principal component analysis (PCA) was performed to extract principal morphological variations (PMVs) of linear combinations of point coordinates.

### Statistical analysi*s*

The correlations between anthropometric parameters: height, weight, BMI, femoral condyle width and axial rotation were tested to understand the study sample. The correlations between anthropometric parameters as the response variables and the PMVs on each principal axis as independent variables were tested to identify the relationships between shape and the anthropometric parameters. They were both tested using the two-tailed Pearson test at a 95% confidence level.

### Quantitative measurement of the extracted PMVs

**Figure 2:**
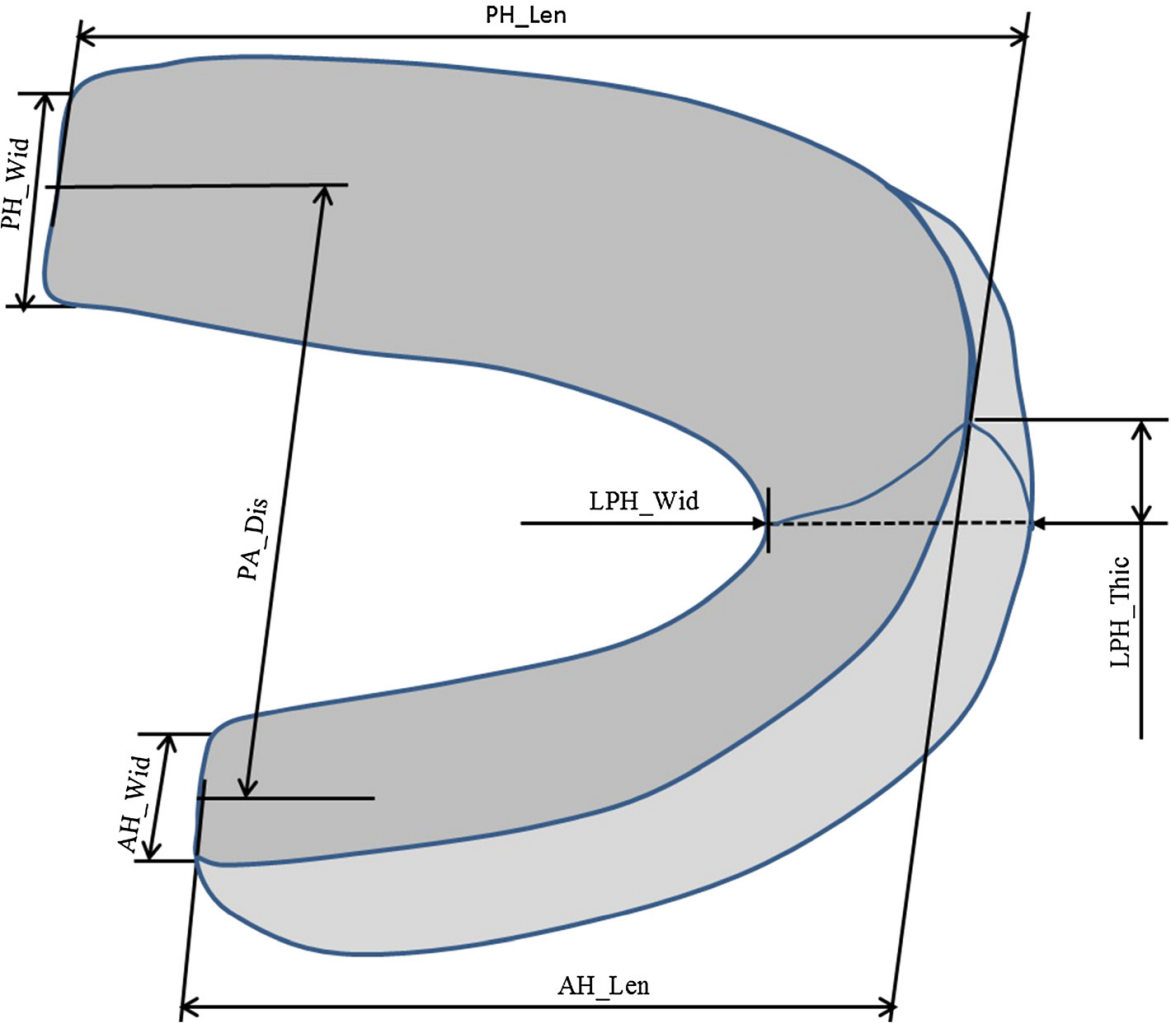
**Seven morphological parameters quantified.** Posterior horn width (PH_Wid): the distance measured between the most posterior and anterior aspects of the posterior horn; anterior horn width (AH_Wid): the distance measured between the most posterior and anterior aspects of the anterior horn; posterior horn length (PH_ Len): the distance measured between the most lateral aspect of the lateral horn and the most medial aspect of the posterior horn; anterior horn length (AH_ Len): the distance measured between the most lateral aspect of the lateral horn and the most medial aspect of the anterior horn; posterior horn to anterior horn distance (PA_Dis): the distance measured between the coronal planes through the middle of the medial aspect of the anterior and posterior horns; lateral peripheral horn thickness (LPH_Thic): the distance measured between the most lateral aspects on the superior and inferior surfaces of the lateral horn; lateral peripheral horn width (LPH_Wid): the distance measured between the most lateral and medial aspects of the lateral horn.

**Figure 3:**
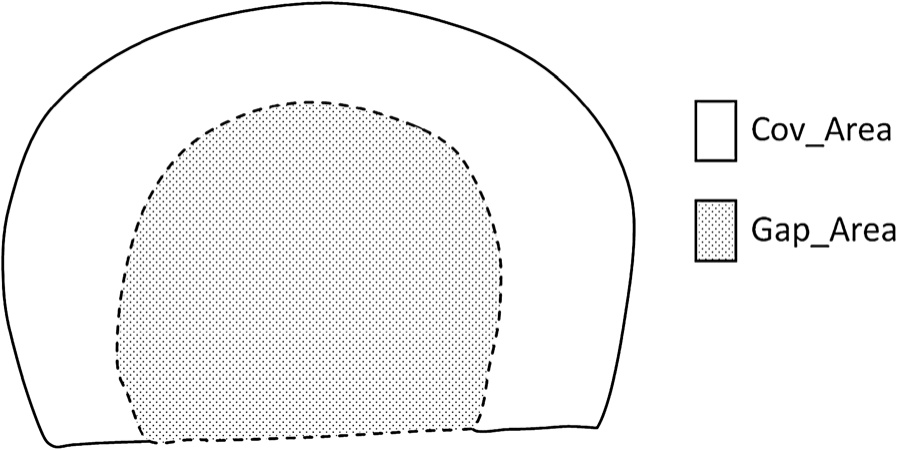
The area covered by the meniscus (Cov_Area) and the gap area between the horns (Gap_Area).

The PMVs extracted from the LM segmentations were used for analysis. Seven quantitative parameters including posterior horn width (PH_Wid), anterior horn width (AH_Wid), posterior horn length (PH_ Len), anterior horn length (AH_ Len), posterior horn to anterior horn distance (PA_Dis), lateral peripheral horn thickness (LPH_Thic) and lateral peripheral horn width (LPH_Wid), were constructed and measured for each PMV to characterise the width and length changes of horns (Figure 2). The reference features to standardise the parameters and the position of the meniscus are shown in Additional file 1. The inferior surface, which is the area covered by the meniscus on the tibial plateau (Cov_Area, Figure 3) and the area of the gap between the interior surfaces of the anterior and posterior horns, which is the area exposed to cartilage (Gap_Area, Figure 3) were measured. The coverage percentage (Cov_Pct) by the LM was calculated as:

$$\mathrm{Cov}\_\mathrm{Pct}=\frac{\mathrm{Cov}\_\mathrm{Area}}{\mathrm{Gap}\_\mathrm{Area}+\mathrm{Cov}\_\mathrm{Area}}$$

The superior surface of the LM, which is the contact area with the femur (Con_Area) was also measured, and the total contact area of the knee joint (Tcon_Area) was calculated as:

Tcon_Area=Gap_Area+Con_Area

To understand the relationship between the openness of the horns and the horn lengths, the ratio of horn distance to the horn lengths (RDL) was calculated as:

$$\mathrm{RDL}=\frac{\mathrm{PA}\_\mathrm{Dis}}{\frac{1}{2}\left(\mathrm{PH}\_\mathrm{Len}+\mathrm{AH}\_\mathrm{Len}\right)}$$

For each PMV, the score on the corresponding principal axis in the SSM was varied from $-2\sqrt{\lambda }$ to $+2\sqrt{\lambda }$ , (where $\sqrt{\lambda }$ is the standard deviation along each principal axis) with the scores on all other principal axes fixed at 0 to monitor the effect of each PMV upon the mean model in the SSM. The change rates of the quantitative parameters were calculated as:

$$\mathrm{\Delta f}=\frac{f_{\mathrm{nMode}+}-f_{\mathrm{nMode}-}}{f_{\mathrm{mean}}}$$

In which *f*_*mean*_ is the quantitative parameter value of the model with a score of 0; *f*_*nMode* +_ and *f*_*nMode* −_ are the parameter values of the model with a score of $+2\sqrt{\lambda }$ and $-2\sqrt{\lambda }$ respectively.

## Results

Weight is correlated with height, and BMI is correlated with weight. Height and weight are correlated with femoral condyle width. These results are as expected (Table 1).

**Table 1:** Results of correlation tests between anthropometric factors

**Height**	**Weight**	**Body mass index**	**Femoral condyle width**
***P *** **( *r *)**	***P *** **( *r *)**	***P *** **( *r *)**	***P *** **( *r *)**
**95% CI**	**95% CI**	**95% CI**	**95% CI**
	<0.001* (0.725)	0.589 (0.079)	<0.001* (0.795)
	0.560, 0.835	---------------	0.663, 0.879
			<0.001* (0.740)	<0.001* (0.764)
		0.581, 0.844	0.617, 0.860	
				0.019 (0.331)
			---------------	

*Statistically significant.

**Figure 4:**
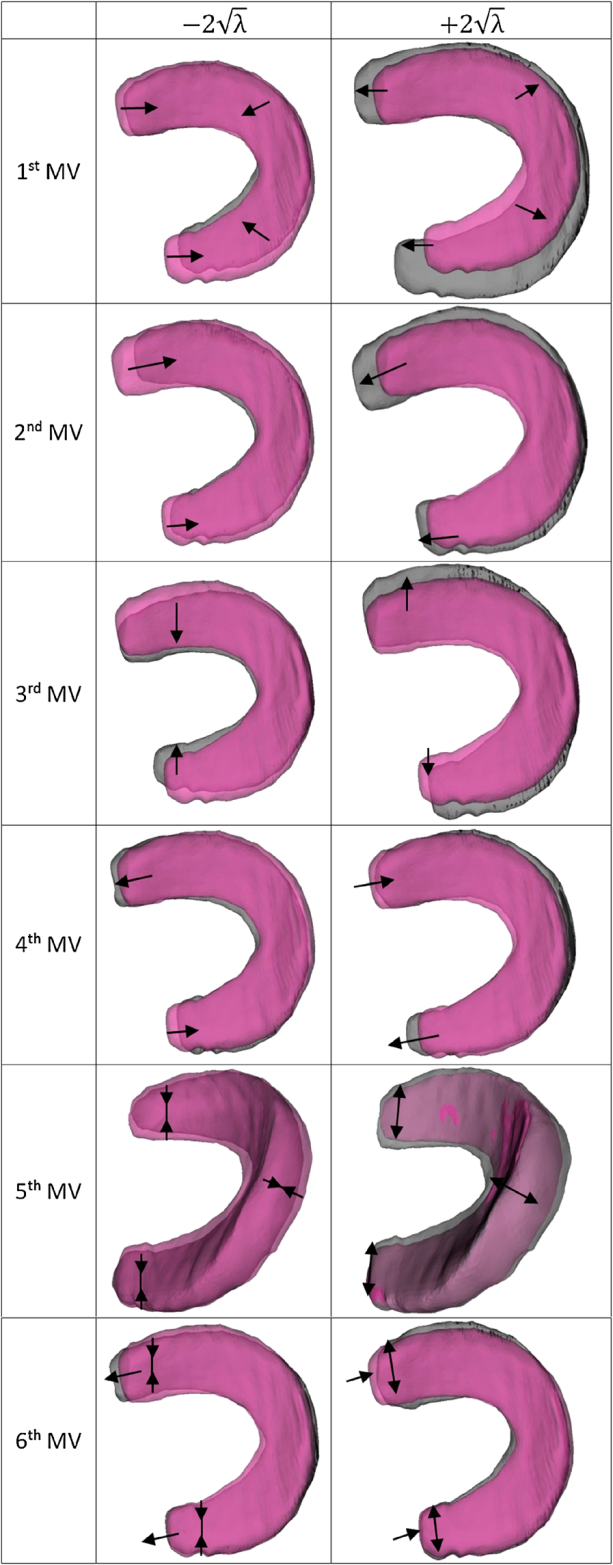
**First six principal morphological variations.** Mean model shown in pink.

The first six PMVs contribute more than 90% of the total variation in meniscal shape for the sample of OAI control subjects. As a result the first six linearly independent PMVs were studied. The change rates of the quantitative parameters and significant features of the first six PMVs are listed in Table 2. Visualisation of the first six PMVs with score of $-2\sqrt{\lambda }$ to $+2\sqrt{\lambda }$ are shown in Figure 4.

**Table 2:** Change rates of quantitative measurements

	**∆Cov_Area**	**∆Cov_Pct**	**∆PH_Wid**	**∆AH_Wid**	**∆PA_Dis**	**∆PH_Len**	**∆AH_Len**	**∆LPH_Thic**	**∆LPH_Wid**	**∆RDL**	**∆Con_Area**	**∆Tcon_Area**	**Significant feature**
	**(%)**	**(%)**	**(%)**	**(%)**	**(%)**	**(%)**	**(%)**	**(%)**	**(%)**	**(%)**	**(%)**	**(%)**	
1^st^ PMV	54.2	-13.1	3.4	5.7	15.5	42.0	35.1	10.2	16.3	-22.2	56.7	50.9	Size
2^nd^ PMV	38.3	9.1	28.0	32.3	-9.2	22.0	22.6	4.8	3.9	-12.3	35.9	37.6	Horn Lengths
3^rd^ PMV	18.7	-14.5	25.8	36.6	33.6	-11.4	-14.4	12.6	1.4	51.7	17.7	18.7	Horn openness
4^th^ PMV	3.2	-8.6	-31.6	8.1	-2.71	17.4	-2.1	4.3	7.4	9.6	6.9	-3.2	Assemetry
5^th^ PMV	31.2	12.6	36.9	41.0	-10.5	6.5	4.7	61.9	47.2	15.4	8.1	31.2	Horn widths
6^th^ PMV	7.05	2.1	15.1	30.9	4.1	-10.3	-4.8	13.5	9.3	11.0	7.2	4.5	Horn thickness

Quantitative measurements include: posterior horn width (PH_Wid), anterior horn width (AH_Wid), posterior horn length (PH_ Len), anterior horn length (AH_ Len), posterior horn to anterior horn distance (PA_Dis), lateral peripheral horn thickness (LPH_Thic), lateral peripheral horn width (LPH_Wid), cover area (Cov_Area), and coverage percentage (Cov_Pct) on tibial plateau, ratio of the distance between horns to the horn length (RDL), contact area (Con_Area) with the femur, and total contact area (Tcon_Area), which sums the contact area with the femur and the area of exposed cartilage. The 1^st^ and 3^rd^ PMVs were found to correlate with height, weight, femoral condyle width, and body mass index.

Correlations with the first and the third PMVs were found with height, weight, femoral size, and BMI. In general the correlations between the PMVs (size, horn openness) and the anthropometric parameters still exist in each individual gender, although they are not as strong as in the mixed gender pool. The correlations between PMV1 (size) and PMV3 (horn openness) and weight are no longer significant in the female group (Table 3). The axial rotation in the 50 subjects is 7.07° ± 7.12° (mean ± standard deviation). No correlations were found with axial rotation of the knee. Statistically significant correlations were not found between the anthropometric parameters and the second, or fourth to sixth PMVs.

**Table 3:** Correlation test results between anthropometric factors and principal morphological variations

	**Mixed gender**				**Male**			**Female**	
	***P***	***r***	**95% CI**	***P***	***r***	**95% CI**	***P***	***r***	**95% CI**
				**Height**					
	<0.001*	0.569	0.381, 0.750	<0.001*	0.897	0.777, 0.948	0.012*	0.497	0.192, 0.714
	**Weigh**t								
	<0.001*	0.647	0.450, 0.784	0.046*	0.360	0.026, 0.622	0114	0.321	-------------
**1**^**st **^**PMV (size)**	**Femoral condyle width**								
	<0.001*	0.622	0.416, 0.748	0.003*	0.563	0.279, 0.756	0.040*	0.414	0.089, 0.659
	**Body mass index**								
	0.002*	0.376	0.109, 0.592	0.039*	0.415	0.091, 0.660	0.260	0.208	-------------
**3**^**rd **^**PMV**	**Height**								
	0.003*	0.406	0.144, 0.615	0.039*	0.416	0.092, 0.660	0.012*	0.546	0.256, 0.746
	**Weight**								
	0.025*	0.312	0.083, 0.510	0.038*	0.417	0.093, 0.661	0.056	0.490	-------------
	**Femoral condyle width**								
	0.019*	0.531	0.236, 0.736	0.054	0.210	-------------	0.046*	0.331	0.070, 0.601
	**Body mass index**								
	0.647	0.006	-------------	0.563	0.121	-------------	0.889	-0.030	-------------

*: statistically significant.

## Discussion

Morphological data on the LM were captured In this study, and were analysed using statistical shape-modelling techniques. The correlation between the first PMV (corresponding predominantly to the size of the meniscus) and the anthropometric parameters of height, weight and condylar width of the knee indicates that when the size of the subject increases, the total contact area increases and the area of the tibia covered by the LM increases as a result of wider and longer horns. However, the gap area between the horns also increases, which causes the coverage percentage of the LM on the tibial plateau to decrease. The correlation between the third PMV (corresponding predominantly to horn openness) and these anthropometric parameters also reveals that when the size of the subject increases, the meniscus becomes more open. Combining the information extracted from the 1st and 3rd PMVs suggests that when the size of the subject increases and more bodyweight has to be transmitted across the knee during regular daily activities, although the actual area covered by the LM on the tibial plateau and the contact area of the LM with the femur increase, the gap area between the horns also increases. This is accentuated by the opening of the horns. In larger subjects, when the knee is required to bear greater loads, the larger total contact area allows the knee joint to maintain low-contact stresses, but the percentage of the area covered by the LM is actually reduced, which implies that the capability of mitigating articular surface contact-stresses could be weakened and greater stresses may develop in the cartilage. This might explain why higher risks of meniscal injuries, knee injuries and OA have been found to be correlated with increased bodyweight [23-26] and height [22].

**Figure 5:**
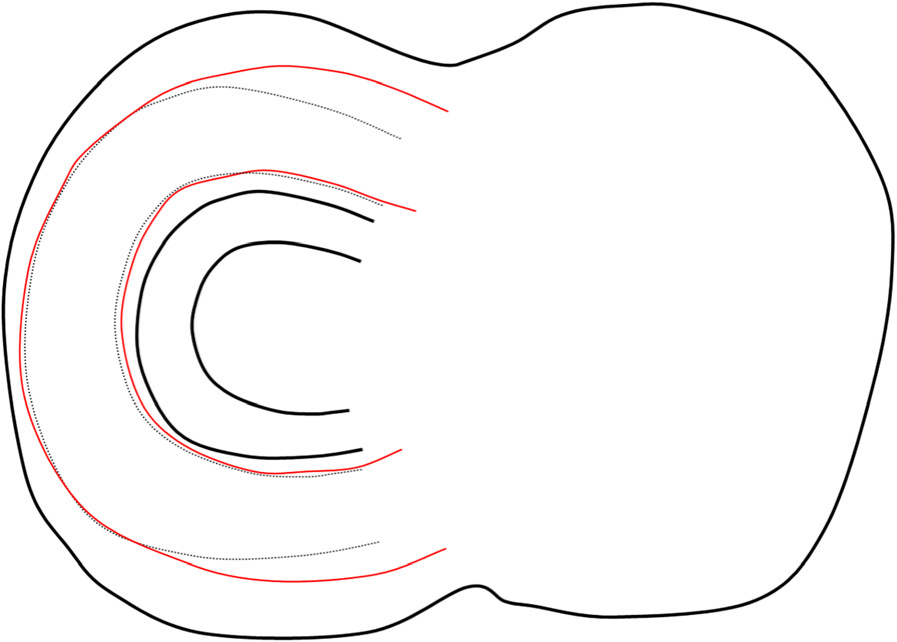
**Superior views of the sketches of the lateral meniscus.** When the size of the subject increases and the tibial plateau becomes larger in size, the mean model (black lines) changes size uniformly (black dotted lines) and according to the first and third principal morphological variations of the statistical shape model (red lines).

The correlations between the PMVs and the anthropometric parameters reveal that when the size of the subject increases, the horns of the LM become proportionally longer and wider (Figure 5). The elongation of the horns can be seen in Table 2 for the first PMV (size of the meniscus), where the ratio between the distance of the horns and the lengths of the horns (∆RDL) is decreased when the distance between the horns (∆PA_Dis) is increased. The increased width of the horns can be seen in Table 2 for the third PMV (∆PH_Wid and ∆AH_Wid). Therefore, the reduced coverage of the tibial plateau is compensated by the variance of the shape. This finding suggests that although larger people generally could have more forces distributed by the articular cartilage in the knee, the shape of the LM appears to compensate for this by having proportionally longer and wider horns. One could hypothesise that wider horns are better able to transmit the circumferential stresses [33] due to higher loading. Secondly, the longer horns would enable the meniscus to translate more due to knee joint rotation and thus be more optimised in terms of location on the tibial plateau. This further supports the hypothesis drawn from previous long-term follow-up studies of total or partial meniscectomy cases and *in vitro* contact area and pressure studies [7,8] that the meniscus has an important role in force transmission across the knee.

Meniscal size, which is a key factor in meniscus transplantation, has been predicted from standard variables including height, weight and gender, according to the literature [17-19]. The results from our study suggest that when using these parameters to predict meniscal size, the variance of the shape accompanied by the change in meniscal size should also be considered to achieve a better distribution of the contact stress in the knee.

The overarching intention of this work is to understand the variations in shape and load-bearing capability of the menisci in both control and OA subjects. To provide a benchmark with which to compare the OA subjects, we analysed the LM from control subjects in this part of the work. During regular daily activities, the knee joints often have to transmit high or repetitive loads. For example, during walking, forces of up to 300% bodyweight can be transmitted across the knee joints [34], and the forces can become four to eight times higher during running [35]. The menisci are thought to bear 40% to 70% of the load across the knee [6]. Our hypothesis is that the meniscus plays an important role in force transmission; therefore, in control subjects the shape of the meniscus could be influenced by the loading, but also should be adapted to cope with the force transmission required during daily activities. The adaptation of knee menisci to prolonged running exercise has been studied in rats by Vailas *et al*. [36]. Significant increases in concentrations of calcium, collagen and proteoglycan were observed in the menisci after the animals were trained extensively for 12 weeks. The thickness of the posterior lateral meniscal horns were also found to be increased. The morphological and biochemical changes seen in the rats were thought to be response and adaptation for the ability of the meniscus to withstand the excessive mechanical stress from prolonged exercise. The relationship between shape and load-bearing capability, however, might be changed in subjects with OA or other joint pathologies, as the tissue properties and loading conditions may have altered the normal functioning of the structures of the joint. Future work will focus on analysing subjects with OA to investigate shape differences between these and control subjects. Further extensions to this study would include the tibial plateau in the shape analysis, as this is likely to be related to the meniscal extrusion, which was believed to a possible effect of the complex interactions among joint tissues and mechanical stresses involved in the OA process in the knee [37].

## Conclusion

Three-dimensional statistical shape modelling was used to extract morphological variations from surface models of the lateral menisci of 50 control subjects obtained from a publically available dataset. The morphological variations and anthropometric parameter analyses show that when the size of the subject increases and more bodyweight is required to be regularly transmitted across the knee, the coverage percentage of the meniscus is reduced, which suggests that there would be an increase in the load the cartilage is required to transmit. However, this reduced coverage percentage is at least partially compensated by the proportionally longer and wider meniscal horns.

## Abbreviations

AH_Len: anterior horn length; AH_Wid: anterior horn width; BMI: body mass index; Con_Area: the superior area of the meniscus that contacts with the femur; Cov_Area: the area covered by the inferior surface of the meniscus; Cov_Pct: the coverage percentage; Gap_Area: the area of the gap between the interior surfaces of the anterior and posterior horns; LM: lateral meniscus; LPH_Thic: lateral peripheral horn thickness; LPH_Wid: lateral peripheral horn width; MR: magnetic resonance; OA: osteoarthritis; OAI: Osteoarthritis Initiative; PA_Dis: posterior horn to anterior horn distance; PCA: principal component analysis; PH_Len: posterior horn length; PH_Wid: posterior horn width; PMV: principal morphological variation; RDL: ratio of the distance between horns to the horn length; SSM: statistical shape model; Tcon_Area: the total contact area of the meniscus with the femur.

## Competing interests

All authors state that they have no competing interests.

## Authors’ contributions

Conception and experimental design: KYZ, AMJB, AEK. Data collection and interpretation: KYZ, AEK. Drafting of manuscript: KYZ. Revising the article critically for important intellectual content: KYZ, AEK, CRD, AMJB, DR. All authors read and approved the final manuscript.

## Supplementary Material

Additional file 1 Reference points and planes that were used as reference features to quantify the extracted morphological variations. Click here for file
