# Supplementary material for: The relationship between lateral meniscus shape and joint contact parameters in the knee: a study using data from the Osteoarthritis Initiative
Source: Arthritis Res Ther. 2014 Jan 28;16(1):R27. doi: 10.1186/ar4455 (PMC3978753; doi:10.1186/ar4455)
Supplement: Additional file 1 — Reference points and planes that were used as reference features to quantify the extracted morphological variations. [file ar4455-S1.docx]

**Additional file** Reference points and planes that were used as reference features to quantify the extracted morphological variations.


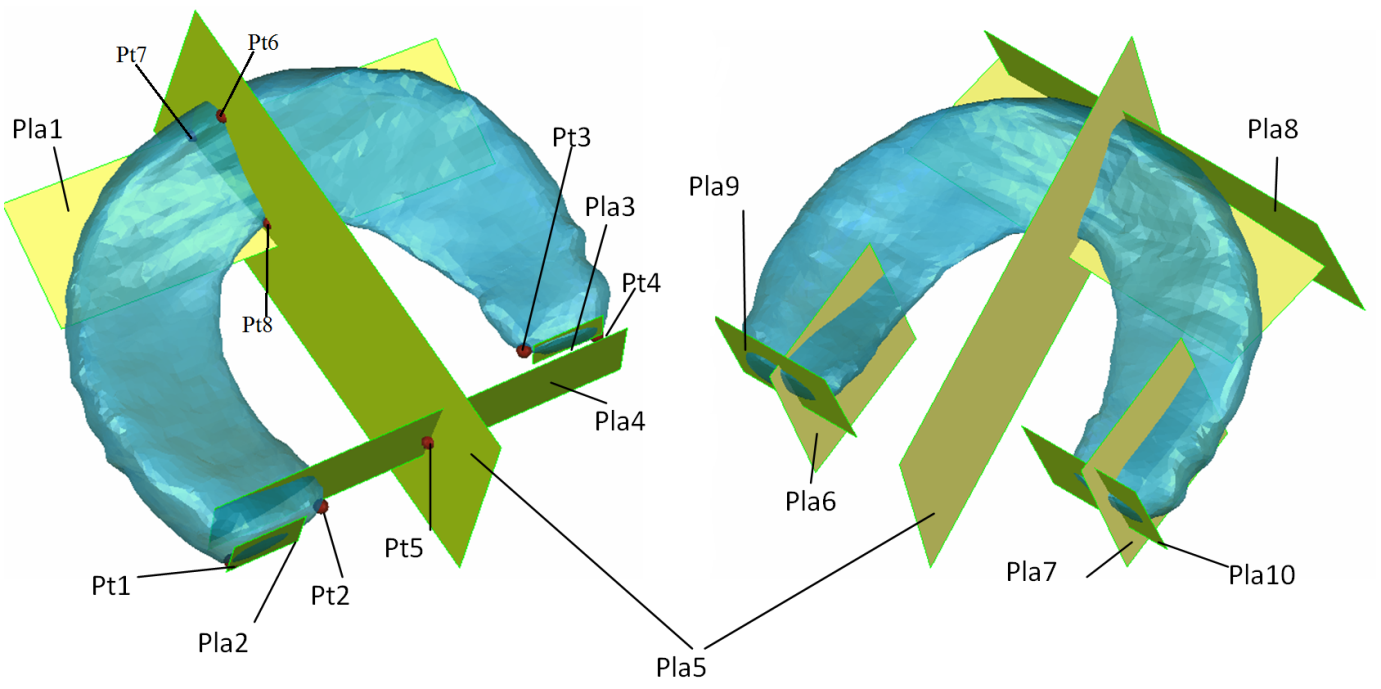


| Parameters used to quantify the meniscus | | |
| --- | --- | --- |
| Posterior horn width (PH_Wid) | | The distance measured between Pt1 and Pt2. |
| Anterior horn width (AH_Wid) | | The distance measured between Pt3 and Pt4. |
| Posterior horn length (PH_ Len) | | The distance measured between Pla8 and Pla9. |
| Anterior horn length (AH_ Len) | | The distance measured between Pla8 and Pla10. |
| Posterior horn to anterior horn distance (PA_Dis) | | The distance measured between Pla6 and Pla7. |
| Lateral peripheral horn thickness (LPH_Thic) | | The distance measured between Pt6 and Pt7. |
| Lateral peripheral horn width (LPH_Wid) | | The distance measured between Pt7 and Pt8. |
| Reference points and planes used to standardise the quantitative parameters and the position of the meniscus | | |
| Pt1 | The most posterior point on the medial superior border of the posterior horn. | |
| Pt2 | The most anterior point on the medial superior border of the posterior horn. | |
| Pt3 | The most posterior point on the medial superior border of the anterior horn. | |
| Pt4 | The most anterior point on the medial superior border of the anterior horn. | |
| Pt5 | The centroid point of pt1, pt2, pt3 and pt4. | |
| Pla1 | The plane fit on the inferior surface. | |
| Pla2 | The plane fit on the medial aspect of the posterior horn. | |
| Pla3 | The plane fit on the medial aspect of the anterior horn. | |
| Pla4 | The average plane of Pla2 and Pla3. | |
| Pla5 | The plane perpendicular to plat1 and plat4, through Pt5. | |
| Pt6 | The most lateral point on the intersection curve between the superior surface and Pla5. | |
| Pt7 | The most lateral point on the intersection curve between the inferior surface and Pla5. | |
| Pt8 | The most medial point on the intersection curve between the superior surface and Pla5. | |
| Pla6 | The plane parallel with Pla5 through the mid-point of Pt1 and Pt2. | |
| Pla7 | The plane parallel with Pla5 through the mid-point of Pt3 and Pt4. | |
| Pla8 | The plane parallel with Pla4 through the mid-point of Pt6 and Pt7. | |
| Pla9 | The plane parallel with Pla4 through the mid-point of Pt1 and Pt2. | |
| Pla10 | The plane parallel with Pla4 through the mid-point of Pt3 and Pt4. | |
